# A Geometrical Approach for Automatic Shape Restoration of the Left Ventricle

**DOI:** 10.1371/journal.pone.0068615

**Published:** 2013-07-19

**Authors:** May-Ling Tan, Yi Su, Chi-Wan Lim, Senthil Kumar Selvaraj, Liang Zhong, Ru-San Tan

**Affiliations:** 1 Geometrical Modelling, Institute of High Performance Computing, A*STAR, Singapore, Singapore; 2 Cardiac Mechanics Engineering and Physiology Unit, National Heart Centre Singapore, Singapore, Singapore; 3 Department of Cardiology, National Heart Centre Singapore, Singapore, Singapore; University of Minnesota, United States of America

## Abstract

This paper describes an automatic algorithm that uses a geometry-driven optimization approach to restore the shape of three-dimensional (3D) left ventricular (LV) models created from magnetic resonance imaging (MRI) data. The basic premise is to restore the LV shape such that the LV epicardial surface is smooth after the restoration and that the general shape characteristic of the LV is not altered. The Maximum Principle Curvature (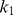) and the Minimum Principle Curvature (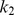) of the LV epicardial surface are used to construct a shape-based optimization objective function to restore the shape of a motion-affected LV via a dual-resolution semi-rigid deformation process and a free-form geometric deformation process. A limited memory quasi-Newton algorithm, L-BFGS-B, is then used to solve the optimization problem. The goal of the optimization is to achieve a smooth epicardial shape by iterative in-plane and through-plane translation of vertices in the LV model. We tested our algorithm on 30 sets of LV models with simulated motion artifact generated from a very smooth patient sample, and 20 *in vivo* patient-specific models which contain significant motion artifacts. In the 30 simulated samples, the Hausdorff distances with respect to the Ground Truth are significantly reduced after restoration, signifying that the algorithm can restore geometrical accuracy of motion-affected LV models. In the 20 *in vivo* patient-specific models, the results show that our method is able to restore the shape of LV models without altering the general shape of the model. The magnitudes of in-plane translations are also consistent with existing registration techniques and experimental findings.

## Introduction

Breath-hold cine Magnetic Resonance Imaging (MRI) is an advanced imaging technique for cardiac morphological and functional assessment in clinical practice. While conventional methods of evaluation are based on MRI images, several recent methods [1], [2], [3] have been developed to utilize three-dimensional (3D) models reconstructed from the MRI data. A 3D model of the LV provides a more comprehensive and accurate description of the ventricular shape and function as properties can be extracted from a combination of in-plane and out-of-plane information, as compared to analysis done on 2D methods. This has a direct impact on the evaluation of LV chamber properties such as its chamber volume, local wall curvature, myocardial wall thickness and wall stress. In our prior study, we have compared the results obtained by conventional 2D methods and 3D methods [2] and found that 3D-based quantification of regional wall stress provides more precise evaluation of cardiac mechanics.

However, factors such as respiration and patient movement contribute to misalignments in the MRI data which results in inaccuracies in the 3D models. MRI data acquired over different breath-hold positions also induce errors in the reconstructed models. [4] aims to correct for misaligned cardiac anatomy, caused by differing breath-hold positions, in multi-slice short-axis (SA) images, by rigidly registering stacks of two slices to a high-resolution 3D MR axial cardiac volume. Existing registration methods also include multi-modal dynamic cardiac image registration [5], external skin marker-based techniques [6], landmark-based techniques [7] and thorax surface-based techniques [8]. A number of cardiac image registration methods are reviewed in [9]. They are categorized into the geometric image feature approach and voxel similarity measure approach. Recently, some post-processing methods have also been proposed. The method proposed by Elen et al. [10] demonstrates the use of constrained optimization, on the assumption that the similarity of gray values at the intersection lines of different slices is higher when the relative positioning of the slices is correct than when the slices are misaligned.

The main dilemma of using an image registration approach to restore the shape of the LV is that errors induced by motion are already embedded in the images. While multi-view image registration techniques could potentially reduce such errors, these methods are essentially using data which contain error for self-correction. In our work, we make use of morphological knowledge of the LV to drive the shape restoration. Instead of using image-based parameters, such as gray values, our LV shape restoration method is based on geometrical consideration. The basic premise is that the LV epicardial surface must be smooth after the restoration, which is a reasonable assumption based on observed morphology, that the myocardial surface tension or force of the LV wall forces the hearts to minimize the wall surface area, creating a smooth epicardial surface. In addition, the general shape of the LV cannot be lost in the process such as its skewness and asymmetrical configuration. The Maximum Principle Curvature 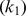 and Minimum Principle Curvature 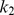 of the LV epicardial surface are used as the geometric measure to quantitate the local shape characteristics.

**Figure 1:**
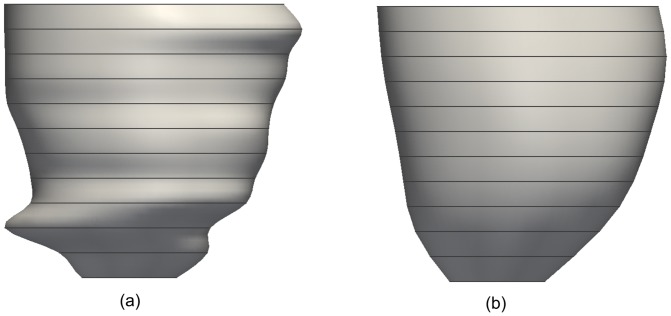
Three-dimensional left ventricle mesh models. (a) with motion artifacts, and (b) desired result after shape restoration.

Figure 1(a) shows a reconstructed 3D LV mesh model from MRI data containing motion artifacts. We aim to achieve a smooth LV mesh as shown in Figure 1(b) after shape restoration. This is achieved by a dual-resolution semi-rigid deformation, followed by a free-form geometric deformation process. The semi-rigid deformation process involves shifting of the myocardium contours by translating each slice in the in-plane direction (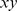-plane), while the free-form deformation process involves translating each individual vertex of the LV model in the 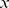-, 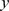
- and 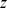-directions. We formulate a smoothness objective function based on 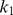 and 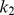, and solve the problem using a limited-memory quasi-Newton optimization algorithm, L-BFGS-B [11]. The L-BFGS-B algorithm is an adaptation of the BFGS algorithm with limited matrix update and it is adept at solving multivariate nonlinear bound constrained optimization problems. This paper is organized as follows: First, we provide detailed explanation of the methodology of our LV shape restoration algorithm. Next we describe the experiments done on the 30 simulated samples and the 20 *in vivo* patient-specific models to test the performance of the algorithm, followed by a discussion on the implications of the experimental results, and finally conclude the paper.

## Methods

The input to our algorithm is an initial 3D LV mesh model 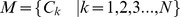 reconstructed from a set of contours {C} representing the myocardial borders delineated from the SA-planes, such that N is the total number of contours. Each contour 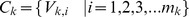 consists of a set of closed connected vertices {V} where 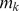 is the total number of vertices in the 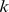-th contour. The convention used is such that the SA-slices are parallel to the 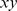-plane; the contours are arranged from the apex to basal region in increasing z-values; and the vertices in the contours are cyclic (i.e., 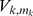 is connected to 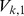, since they represent a closed connected curve).

### Shape Quantification Using Principle Curvatures 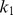 and 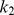


In this section, we briefly outline the formulation of 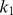 and 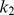. In order to interrogate the geometrical properties of the LV epicardial surface mesh, we use a quadric fitting method to approximate the underlying geometry at every vertex of the mesh. A quadric surface S in 3D space can be expressed in the parametric form
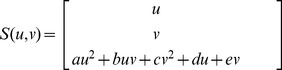
(1)where 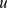 and 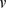 are the surface parameters and 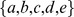 are the quadric coefficients. To fit 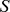 at a vertex 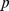, we select a neighborhood around 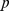 which represents the region over which 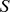 is to be fitted. The extent of this neighborhood is quantified by a 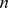-ring value. The quadric coefficients of 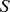 are then obtained by solving a system of linear equations associated with the 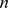-ring neighborhood using a least square method [12]. The surface 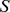 approximates the local geometry in the vicinity of a point 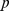 on the 3D mesh model. In differential geometry, the curvature of a surface 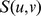 at a point 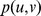 is evaluated with respect to a normal section. This is done by constructing a plane 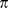 such that it passes through the unit surface normal 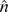 and unit tangent vector in the direction of 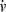 (where 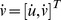). The intersection of 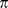 with 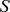 results in a curve called the normal section. The normal curvature 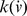 can be evaluated by
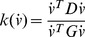
(2)where 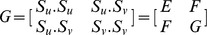 and 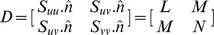 are the first and second fundamental matrices of the surface, respectively. The unit surface normal can be calculated by



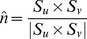
(3)In terms of the quadric coefficients, the equations to calculate 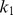 and 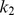 are
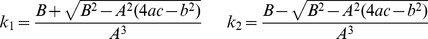
(4)where 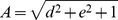 and 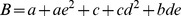.

The value of 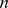-ring used in the quadric fitting affects the value of 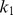 and 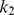 because it determines how sensitive the method is to the effect of geometrical variation. With a bigger 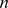-ring value, shape of the surface over a larger extent is interrogated. This takes into account the general variation of the shape, ignoring the high frequency variation in the geometry. With a smaller 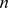-ring value, the shape of the surface over a localized region is inspected. This captures the inter-slice variations in shape.

### Shape-based Optimization Objective Function

The basic premise of the shape restoration is the assumption that the LV epicardial surface is smooth. From a shape characterization perspective, this implies that it should have minimum local concavity. The objective is then to find the optimal modifications in the LV model such that its total concavity is at a global minimum. Computationally, we can calculate the maximum and minimum principle curvatures (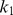 and 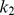, respectively) for every point on the LV epicardial surface mesh to assess the amount of concavity or convexity of the surface. When 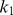 or 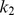 is negative, it implies that the surface at which a point lies on is concaved. Therefore, to minimize concavity, the objective function 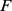 only takes into account the summation of 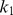 and 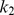 values of all points with negative 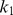 or negative 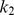, that is,
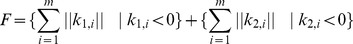
(5)where 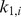 is the maximum principal curvature and 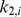 is the minimum principal curvature at vertex 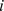 and 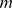 is the total number of vertices in the epicardial surface mesh.

This non-linear objective function can be minimized using the L-BFGS-B algorithm [11] which is adept at solving multivariate nonlinear bound constrained optimization problems. It is based on the gradient projection method and uses a limited-memory BFGS matrix to approximate the Hessian of the objective function. The algorithm does not store the results from all iterations but only a user-specified subset. Its advantage is that it makes simple approximations of the Hessian matrices which are still good enough for a fast rate linear convergence and requires minimal storage [11]. This will result in geometrical kinks being smoothed out but not at the expense of creating more kinks in other locations. The result is an improvement in the overall smoothness of the LV shape.

In order to maintain the overall shape characteristic of the LV model, such as its skewness and asymmetrical configuration, the optimization is performed in a dual-resolution semi-rigid geometrical deformation whereby the LV model is modified from a global and regional consideration, followed by a freeform geometrical deformation process for localized optimization.

### Dual-resolution Semi-rigid Geometric Deformation

**Figure 2:**
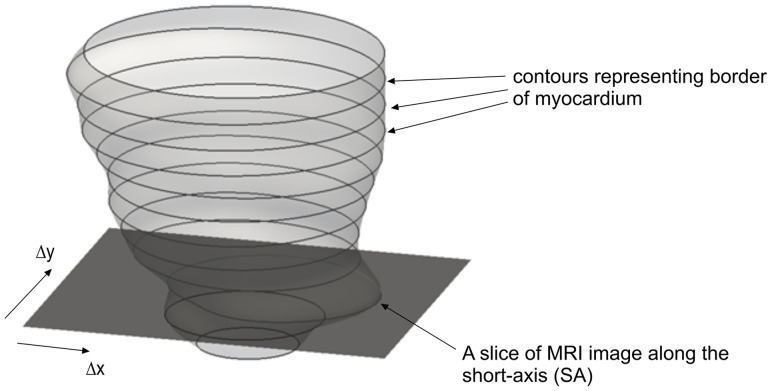
Shape Restoration of LV mesh in the x- and y-directions. Modifying shape of 3D LV epicardial surface mesh by translating the contours on SA slice in x- and y-directions.

As the 3D LV models are reconstructed from contours of the myocardial borders, the first stage of the shape restoration process works by progressively translating these contours in the plane of their respective SA-slice. Figure 2 illustrates the shifting of the contour on a SA-slice in the in-plane direction. One can view this as a semi-rigid mesh modification since we are keeping the shape of the contours constant while shifting their position. For every slice, a centroid 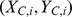 is calculated by averaging the 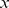
- and 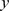-coordinates of all the points from that particular slice, where 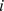 is the slice index. The optimization algorithm, L-BFGS-B, will solve for the optimal 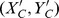 for all the slices to satisfy the objective function in Equation (5).

To set up the optimization problem, we can write Equation (5) as 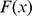 with 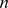 variables, such that 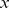 contains the centroid coordinates 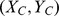 of the contours on the SA-slices, i.e
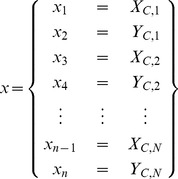



To retain the skewness and asymmetry of the dataset, we fix the centroid coordinates of the top and bottom SA slices as boundary conditions, i.e. 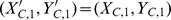 and 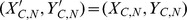. Hence, the number of variables in the optimization problem is 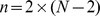. Each of the variables 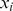 in 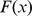 is subjected to the bounded-constraints
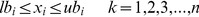
(6)where 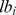 and 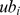 are the lower and upper bounds of 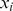, respectively. In this work, the variables are constrained to translate within a bound of 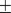 20 mm. This value is consistent with what was observed experimentally [13] (maximum displacement recorded upon inhalation is 23.5 mm). The constraint on the translation in the 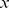-direction is
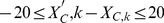
(7)where 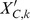 is the solution and 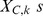 is the initial 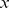-coordinate of the centroid position of the 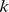-th contour. Similarly, the constraint on the translation in the 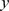-direction is
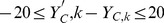
(8)where 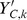 is the solution and 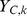 is the initial 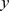-coordinate of the centroid position of the 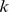-th contour. In addition, the gradient 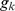 associated with each variable 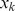 must also be defined such that



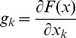
(9)Since 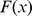 is in a non-analytical form, we need to approximate 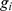 using finite differences. In this work, we use the forward difference method to approximate the gradient, i.e.,
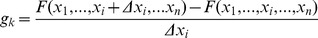
(10)where 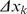 is a small increment in 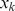.

In order to retain the general variation of the LV shape, we perform the optimization in dual stages – a global stage and a regional stage. As the objective function in the optimization is computed using a 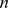-ring setting, we employ that to determine the nature of the shape characterization of the LV. In general, a large 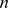-ring will result in shape characterization from a global perspective while a smaller 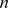-ring will result in a regional/local shape characterization.

In the global stage, the value of 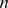-ring selected is adaptive to the sampling resolution of the LV model. For our application, it is half the number of MRI stacks constituting the whole LV. However, the 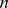-ring selected cannot be too huge as computation time increases with the value of 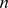-ring. Therefore, we enforce that
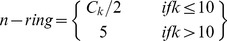
(11)


**Figure 3:**
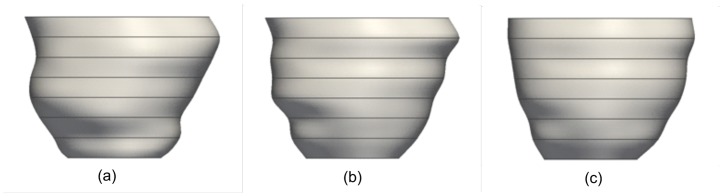
Shape restoration using dual resolution semi-rigid deformation. (a) original mesh with motion artifact, (b) intermediate mesh after optimization using n-ring  = 5, and (c) final mesh after optimization using n-ring  = 2.

In the example shown in Figure 3(a), it shows an original mesh with motion artifact, where the 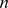-ring selected is 4 due to it having 8 MRI stacks. When 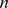-ring  = 4, 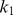 and 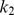 are calculated by taking into account points from 4 layers above and below the current SA slice, and 4 points to the right and left of the point of interest. All the slices will shift to minimize the objection function in Equation (5). Next, to further minimize surface concavity over a smaller region of consideration, the intermediate mesh (updated with the previously obtained solution using 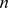-ring  = 4) is subjected to a second pass of optimization using a fixed 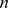-ring  = 2. This second pass is essential to further minimize the concavity over a localized region. The results from setting 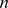-ring value  = 4 and then 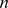-ring  = 2 are shown in Figure 3(b), intermediate mesh after optimization using 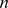-ring  = 4 and Figure 3(c), final mesh after optimization using 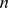-ring  = 2. As observed, the LV smoothness was restored.

### Free-form Geometric Deformation

In the previous section, we discussed a dual-resolution semi-rigid deformation process, whereby only translational displacement of the SA-slices is considered. However, in actual fact, motion artifacts are also generated by motions in and out of the SA-planes. Therefore, in the next stage, a local free-form geometric deformation is performed. Here, the output from the semi-rigid geometric deformation process forms the input to the free-form deformation process. The shape restoration is done by progressively translating each individual vertex of the LV model in the 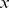-, 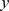
- and 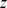-directions. Using the same assumption that the LV epicardial surface is smooth, our objective is to find the optimal translations in the 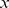-, 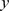
- and 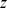-directions for each individual vertex such that the total concavity of the whole LV is at its global minimum. The same objective function 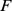 in Equation (5) is used and after the constraints on the translation distance 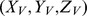 are set, the L-BFGS-B algorithm is used to solve for the optimal translations of all the vertices of the LV mesh. The optimal 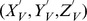 are then used to update the mesh. To set up the optimization problem, we can write Equation (5) as 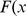) with 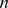 variables, such that 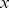 consists of the vertex coordinates 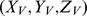 of the mesh, i.e.,
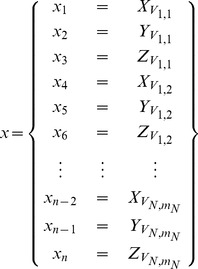



To retain the skewness and asymmetry of the dataset, we fix all the vertex coordinates of the top and bottom SA slices as boundary conditions, i.e. 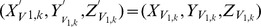 for all 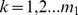 and 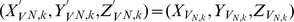 for all 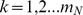. The total number of vertices in the mesh is 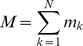. Hence, the number of variables in the optimization problem is 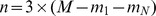. Again, each of the variables 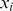 in 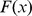 is subjected to the bounded-constraints. The constraints in the 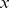
- and 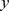-directions are determined by the distance between the vertex and its immediate neighboring vertices on the same slice/contour:
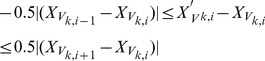
(12)where 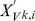 is the solution and 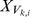 is the initial 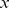-coordinate of the position of vertex 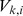. Similarly, the constraint on the translation in the y-direction is



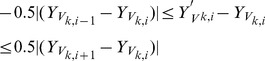
(13)The constraint on the translation in the 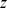-direction is determined by the inter-slice distance such that
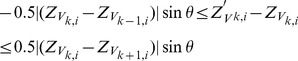
(14)where 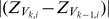 is the original distance between contour 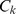 and its lower adjacent contour 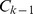; 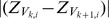 is the original distance between contour 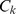 and its upper adjacent contour 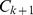; and 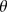 is the angle between the vertex normal and the z-axis.

**Figure 4:**
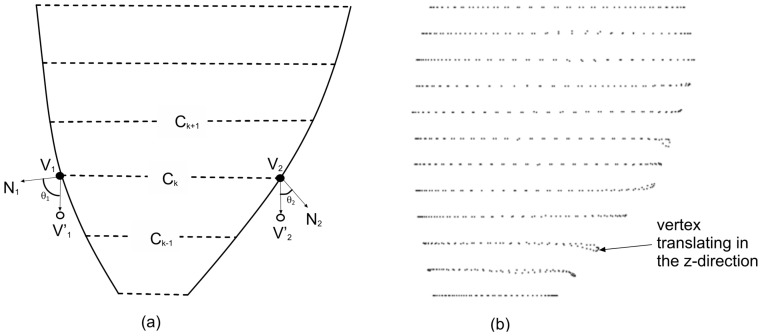
Constraint in the z axis. (a) Angle 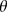 between vertex normal 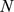 and the 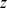-axis, and the impact on volume change due to translation, (b) LV epicaridal surface modified by the free-form deformation process.

In Figure 4(a), we illustrate the impact of 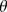 on the volume of the LV mesh with respect to the vertical shift of the vertices. Two vertices 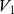 and 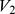 on the same contour 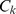 are such that 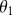
>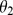. Given the same allowance of vertical shift such that their new positions become 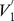 and 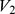, we observed that the deviation of 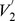 from the original surface of the mesh is greater than 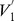, indicating that when 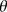 is smaller, the resulting volume change due to the vertical vertex shift is larger. Therefore, the 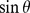 function in Equation (14) is used to constrain the vertices such that if there is greater deviation between the vertex normal from the SA-plane (i.e., 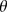 is small), the allowable vertical translation in the 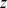-direction will be less. This will prevent unduly large change in the volume of the restored mesh. Figure 4(b) illustrates an LV epicardial surface modified by the free-form deformation process.

**Figure 5:**
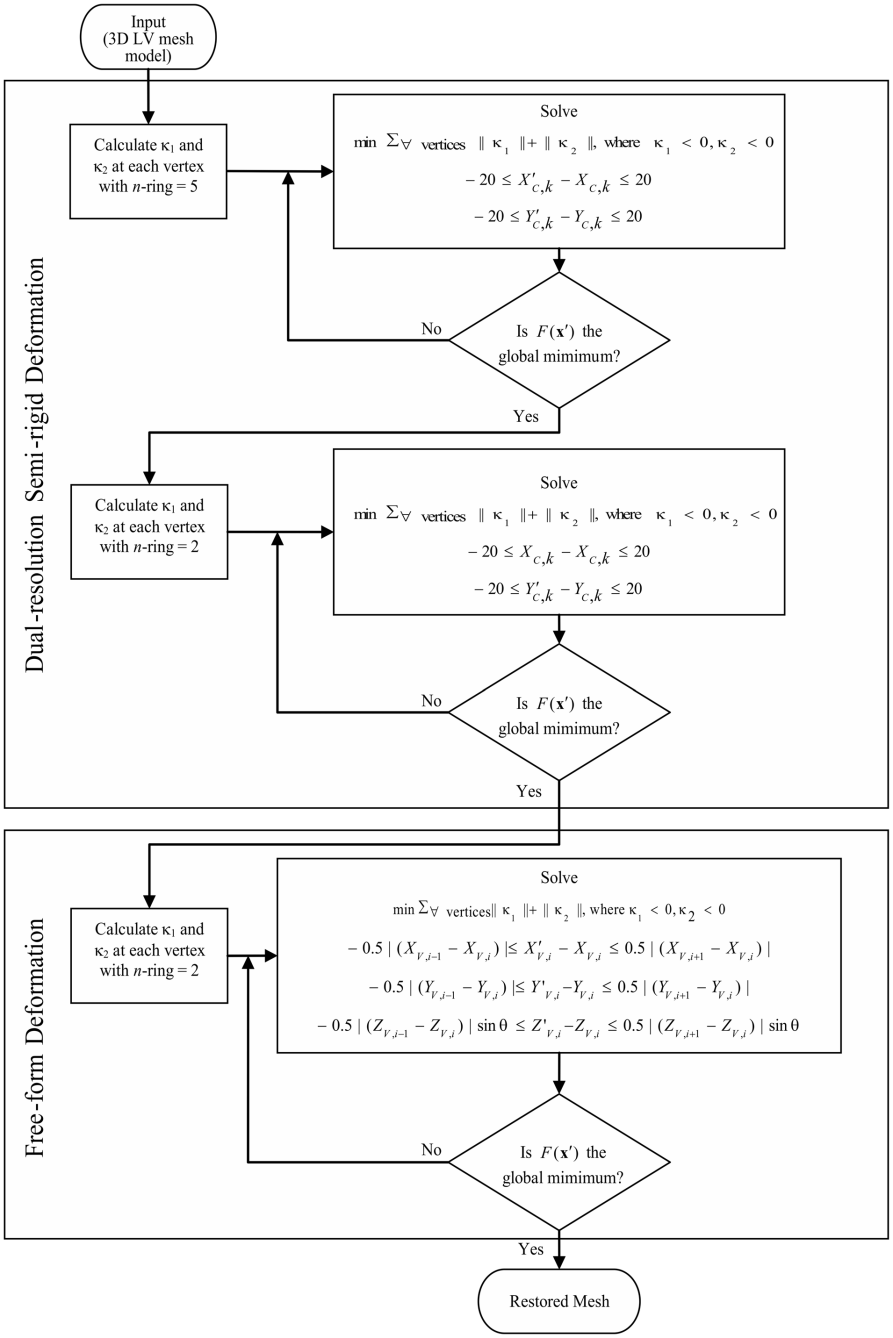
Flow chart of the restoration process. Flow chart of the dual-resolution semi rigid and free-form restoration process.

Figure 5 shows the flow chart of the whole restoration process.

## Results and Discussion

### *In vivo* Patient-specific Models

**Figure 6:**
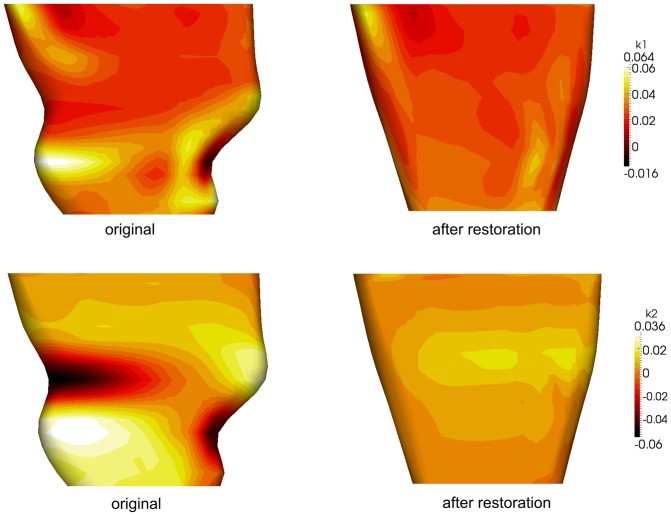
Changes in 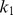 and 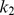 before and after dual and free form geometric restoration. One sample of patient-specific 3D LV mesh model with its 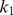 and 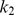 values before and after dual and free form geometric deformation.

**Figure 7:**
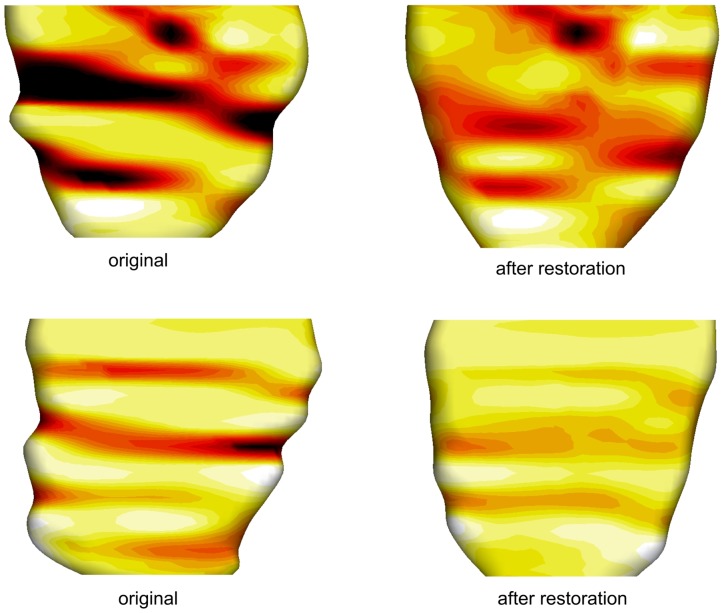
Patient-specific 3D LV mesh model restoration results. Two samples of patient-specific 3D LV mesh model before and after dual and free form geometric deformation.

In this section, we tested our algorithm on 20 patient-specific 3D LV models reconstructed from MRI data containing motion artifacts. This study was approved by the SingHealth Centralised Institutional Review Board (CIRB No: 2009/705/C) for Human Research. All enrolled participants gave written informed consent. The MRI scan was performed using breath-held steady-state free precession technique on a 1.5T Siemens scanner (Avanto, Siemens Medical Solutions, Erlangen). TrueFISP (fast imaging with steady-state precession) MR pulse sequence with segmented k-space and retrospective electrocardiographic gating were used to acquire 2D cine images of the LV in the long-axis (LA) plane, as well as a parallel stack of 2D cine images of the LV in the SA plane, from the LV base to apex (8 mm inter-slice thickness, no inter-slice gap). Each slice was acquired in a single breath hold, with 25 temporal phases per heart cycle. The epicardial borders of contiguous SA-slices were manually delineated by an experienced cardiologist using commercially-available software CMRtools (Cardiovascular Imaging Solution, UK). Both SA- and LA-views were utilized to carry out 3D LV reconstruction at the end-diastole phase. Figure 6 plots the absolute values of 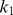 and 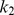 before and after restoration of one of the patient sample. Quantitatively, the absolute values of 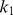 and 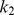 were reduced considerably after the shape restoration. Visually, we observed that the asymmetry of the LV geometry was preserved while the geometrical kinks on the surface were significantly reduced. Figure 7 shows that the shape of 2 other patient samples became smoother and retained their asymmetry after the whole dual and free form geometric deformation process. The results indicated that average contour displacements in the SA-planes were 1.36 mm and 1.30 mm, with a maximum translation magnitude of 8.68 mm and 8.65 mm in the 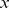
- and 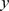-directions respectively. The vertices are further displaced in the free-form deformation by an average of 0.10 mm, 0.11 mm and 0.07 mm, with a maximum translation magnitude of 1.73 mm, 1.95 mm and 2.93 mm in the 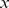-, 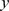
- and 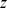-directions, respectively.

As it is difficult to obtain the corresponding ground truth for each of the 20 *in vivo* patient-specific model, we assess the performance of our restoration algorithm by comparing the mean contour displacement values of our method with those of existing image registration techniques [14], as shown in Table 1, whereby the mean translations in the x-direction are 3 mm [15], [16] and y-direction is 3.25 mm [17] and z-direction is 1.5 mm [18]. The maximum experimental translation observed in [13] is 23.5 mm. Our results are observed to lie within the range of existing literatures.

**Table 1:** Overview of Evaluated Cardiac and Thorax Image Registration Methods [9].

Reference	Movement Correction (mm)
[21]	2.19±0.52
[15]	(x,y) 3.0
[14]	2.8±0.5
[22]	2.7
[23]	1.95±1.6
[24]	2.5
[17]	(x)1.23±0.06, (y) 3.25±1.04
[16]	(x) 3.0, (y) 1.6
[25]	(x,y,z) 1.0
[26]	(x,y) 1.7
[8]	(x) 1.9, (y) 2.4
[27]	(x,y) 0.5±0.5
[28]	2.5±1.2
[29]	3.1±1.7
[18]	(x,y,z) 1.5

### Models with Simulated Motion

To further validate the applicability of our method, we study the performance of our method on a set of LV data with simulated motion. This is done by selecting a gold standard to act as a reference or ground truth for comparison. Ten healthy volunteers underwent breath hold cine-MRI scan. The sample with the smoothest epicardial surface is selected as the gold standard. To do this, the respective 3D models of the LV were reconstructed for each of the 10 volunteers and we subject these 3D models to a computational algorithm to assess the quantitative value of the local curvature. In addition, we apply our shape restoration method to these 3D models. The model which yields the minimum concavity and has the least modification in the restoration process is selected as the Ground Truth.

This Ground Truth model is then used as a reference to investigate our algorithm's ability in performing LV shape restoration. We proceed to construct a set of 30 random samples from the ground truth model by translating three randomly selected but consecutive slices to simulate motion artifacts. They are translated within the range of ±20 mm. These 30 randomized samples are then restored using our proposed algorithm and compared against the ground truth model for validation. To evaluate the accuracy of the restoration, we make use of The Metro Software [19] a tool designed to evaluate the difference between two triangular meshes. Metro adopts an approximated approach based on surface sampling and point-to-surface distance computation. The Hausdorff distance evaluated by the Metro Software measures the dissimilarity between two shapes, and it is used to quantify how different the LV mesh is before and after restoration, as compared to the ground truth. From Table 2, we can see that the Hausdorff distance after restoration w.r.t the ground truth model is much smaller than that before restoration, except for Sample 1. This indicates that the restored meshes are more similar to the ground truth after the restoration process. In Sample 1, it is noticed that the Hausdorff distance w.r.t the ground truth model before restoration is just 1.03 mm. This implies that the randomly generated sample did not differ much from the ground truth model. The huge percentage difference of 21% is due to this small Hausdorff distance w.r.t the ground truth model before restoration. In addition to the analysis using Hausdorff distance, we evaluated the restoration using curvedness comparison. We have previously shown that curvedness is an important shape characterization measure for LV models and that it is associated with important LV functions [2]. Table 3 shows the curvedness results of the ground truth and the mean curvedness results of the 30 samples before and after restoration over 16 regions. Table 4 shows the absolute difference in curvedness with respect to the ground truth over 16 regions. The absolute difference in value and percentage of the curvedness with respect to the ground truth after restoration improved significantly for each region, which implies that curvedness value is closer to the ground truth after restoration.

**Table 2:** Hausdorff Distance w.r.t Ground Truth Before and After Restoration.

Sample	Before(mm)	After(mm)	Improvement
1	1.03	1.25	−0.22 (−21%)
2	8.87	3.14	5.73 (65%)
3	8.56	3.18	5.39 (63%)
4	8.49	2.31	6.18 (73%)
5	9.27	3.74	5.53 (60%)
6	12.35	3.56	8.79 (71%)
7	9.38	2.53	6.85 (73%)
8	4.86	1.67	3.19 (66%)
9	11.7	2.37	9.33 (80%)
10	9.38	2.6	6.78 (72%)
11	6.95	3.7	3.25 (47%)
12	6.8	2.8	4.01 (59%)
13	11.3	2.34	8.95 (79%)
14	2.79	1.59	1.20 (43%)
15	8.78	3.65	5.13 (58%)
16	6.52	2.78	3.74 (57%)
17	9.75	3.46	6.29 (64%)
18	10.09	3.32	6.76 (67%)
19	6.7	3.21	3.49 (52%)
20	6.85	2.92	3.93 (57%)
21	10	3.19	6.81(68%)
22	8.35	3.89	4.46 (53%)
23	9.35	2.89	6.46 (69%)
24	6.77	2.39	4.38 (65%)
25	10.81	3.38	7.43(69%)
26	4.87	2.1	2.78 (57%)
27	5.62	2.74	2.88 (51%)
28	8.79	2.26	6.53 (74%)
29	11.25	4.69	6.55 (58%)
30	1.29	1.27	0.02 (1%)

**Table 3:** Curvedness Values of Ground Truth and Mean Curvedness Values of 30 Samples before and after Restoration over 16 Regions.

Region	Ground Truth	Before Restoration(Mean)	After Restoration(Mean)
1	0.0179	0.0213	0.0191
2	0.0178	0.0212	0.0188
3	0.0171	0.0192	0.0174
4	0.0177	0.0206	0.0173
5	0.0203	0.0231	0.0194
6	0.0196	0.0218	0.0199
7	0.0202	0.0248	0.0196
8	0.0187	0.0241	0.0188
9	0.0185	0.0233	0.0187
10	0.0211	0.025	0.0212
11	0.0166	0.0219	0.0167
12	0.0222	0.027	0.022
13	0.0249	0.0281	0.0259
14	0.0285	0.0333	0.0281
15	0.0273	0.0321	0.0273
16	0.0242	0.0281	0.0248
Total	0.3327	0.3948	0.3351

**Table 4:** Absolute Difference and Percentage Difference in Curvedness w.r.t Ground Truth.

Region	Before Restoration(Mean)	After Restoration(Mean)
1	0.0034 (19%)	0.0012 (7%)
2	0.0033 (19%)	0.0009 (5%)
3	0.0021 (12%)	0.0003 (2%)
4	0.0028 (16%)	0.0005 (3%)
5	0.0028 (14%)	0.0008 (4%)
6	0.0022 (11%)	0.0003 (1%)
7	0.0046 (23%)	0.0006 (3%)
8	0.0054 (29%)	0.0001 (0%)
9	0.0049 (26%)	0.0003 (1%)
10	0.0039 (18%)	0.0001 (0%)
11	0.0053 (32%)	0.0000 (0%)
12	0.0048 (22%)	0.0002 (1%)
13	0.0033 (13%)	0.0010 (4%)
14	0.0048 (17%)	0.0004 (1%)
15	0.0047 (17%)	0.0000 (0%)
16	0.0039 (16%)	0.0006 (2%)
Total	0.0621 (19%)	0.0073 (2%)

### Limitations and Challenges

As mentioned in the above section, the limitation of this study is in the validation of the restoration results of the 20 *in vivo* patient-specific models. While we do not have the ground truth for each of the patient-specific model, we compared the results of the mean contour displacements and observed that the values lie within the range reported in existing literatures. In addition, we attempted to account for this limitation by performing an experiment with simulated motion and observed significant improvement in terms of Hausdorff distance and curvedness.

We would also like to highlight that the derivation of the optimization function that drives the shape restoration is based on the observed morphology that the surface tension of the LV wall physically forces the heart to minimize the wall surface area, creating a smooth epicardial surface. However there are questions if this assumption still holds true for severe pathological conditions such as acute myocardial infarction. In a study of ventricular shape after myocardial infarction by Mitchell et al. [20], global LV geometry was assessed on cine angiography by means of a sphericity index. This index is the ratio of LV volume and a sphere with similar circumference. After myocardial infarction, the heart assumes a more spherical conformation (i.e., sphericity index increases), implying an overall diminution of surface concavity. This argument is also supported by calculating the total ventricular force or tension (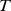) from 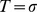 x 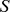, wherein 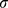 is the ventricular wall stress, and 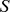 is the surface area. For any given pressure and wall stress 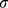, 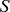 being smallest would offer an optimal energy solution for moving blood. From clinical observation, the ventricles of patients with pathological condition remodel towards a spherical configuration (i.e., for any given ventricular volume, the surface area 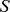 is the smallest when the ventricular geometry is spherical), implying a “smoother” epicardial surface.

## Conclusion

In this paper, we presented an automatic algorithm to restore the shape of a 3D LV mesh model using a geometry-driven optimization approach. The method used an analytical surface fitting method to approximate the geometry of the LV mesh and computed the minimum principal curvature as a quantification of the surface smoothness. Next, a limited memory quasi-Newton algorithm, L-BFGS-B, was used to correct the positions of all SA-slices to achieve an optimal shape with minimal concavity. To retain the overall shape of the LV mesh, such as its asymmetrical configuration, we performed optimization using 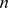-ring to be half the number of contours representing the myocardial borders. Next, to achieve localized smoothing, we performed a second pass of optimization using 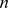-ring  = 2. 30 sets of simulated data and 20 *in vivo* LV epicardial datasets at end-diastole are used as inputs to investigate the performance of our shape restoration algorithm. The results showed that there were significant improvements in the smoothness of the LV mesh both visually and quantitatively (in terms of the magnitude of maximum and minimum principal curvatures). Also, our algorithm was successful in preserving the overall shape of the LV mesh without over smoothing. Our results are also consistent with results of existing image registration techniques. Another 30 samples of data sets generated from a smooth patient set (i.e. Ground Truth), were restored and the difference in Hausdorff distance and curvedness values w.r.t the Ground Truth were smaller after restoration.
